# T cell-mediated tumor killing patterns in head and neck squamous cell carcinoma identify novel molecular subtypes, with prognosis and therapeutic implications

**DOI:** 10.1371/journal.pone.0285832

**Published:** 2023-05-16

**Authors:** Zilu Meng, Lei Zhu, Wanyu Liu, Wenhan Yang, Yudong Wang

**Affiliations:** Department of Maxillofacial Surgery, The First Affiliated Hospital of Guangdong Pharmaceutical University, Guangzhou, China; Concordia University, CANADA

## Abstract

As an important process in cancer immunotherapy, T cell-mediated tumor killing (TTK) enhances the immune response of patients. However, the role of TTK in Head and Neck Squamous Cell Carcinoma (HNSCC) patients still needs further exploration. Therefore, we comprehensively analyzed the gene expression information and clinical characteristics of 1063 HNSCC in five cohorts. Univariate regression, differential expression analysis, and gene mutation profiling were combined to identify the important genes regulating the sensitivity of tumor cells to T cell-mediated killing (GSTTK) in HNSCC. A total of 20 GSTTK were identified as important genes of HNSCC. Patients were divided into C1 and C2 subgroups (TTK patterns) and displayed significant prognostic differences. Patients with C2 subtype had dismal prognosis characteristic compared to C1 subtype in all validation cohorts. Patients with C1 subgroup exhibited robust immune profile and C1 subgroup patients were significantly enriched in metabolically relevant functions. Notably, the multi-omics analysis found that C1 subgroup have higher mutation burden and C2 subgroup patients had significantly higher copy number variation. Drug sensitivity analysis found that multiple first-line chemotherapeutic drugs were more sensitive in patients with subgroup C1. In conclusion, the establishment of GSTTK provides guidance and assistance to clinicians in the personalized management and treatment of HNSCC patients.

## 1. Introduction

According to the 2018 global cancer statistical report, head and neck squamous cell carcinoma (HNSCC) reported more than 600,000 new incidents and nearly 400,000 fatalities annually, which was the 6th leading cause of cancer-related mortality [1]. HNSCC frequently occurs in the oral cavity, oropharynx, hypopharynx, and larynx, which is a multi-organ malignant disease [2]. Patients with HNSCC present dismal overall survival (OS) and high recurrence rate, especially combined with smoking, alcohol consumption, and chronic infection of human papillomavirus, which can increase the risk of HNSCC development and recurrence by tenfold [2–4]. Although the treatment of HNSCC patients has been further improved, the five-year survival rate of patients has not reached 60% [2,5]. The application of surgery, radiotherapy, chemotherapy, targeted therapy, and combination therapy has provided clinicians and patients with more treatment options [6], but these treatments have corresponding limitations (including multiple complications and treatment resistance) [7], and the treatment of HNSCC patients still faces huge challenges. With more in-depth research, it was found that the same treatment modalities have different efficacy for patients, which led to overtreatment and undertreatment in clinical practices [8]. Therefore, reasonable stratification of patients is an urgent issue which will contribute to the refined management and personalized treatment of patients in clinical practice and improve the application efficiency of medical resources.

In recent years, immunotherapy has been widely used in the treatment of solid tumors. Immune checkpoint inhibitors (ICIs) are antibodies against primary immune surveillance escape mechanisms, which are a major cancer therapy and positively impact treatment outcomes in cancer patients [9]. It is believed that ICIs are a promising treatment for HNSCC patients because it is an immunosuppressive disease [10]. In the CheckMate 141 study, researchers found that HNSCC patients treated with nivolumab experienced significantly longer survival than investigator’s choice (IC) patients regardless of PD-1 expression and HPV status (Clinicaltrials.gov: NCT02105636) [11]. Additionally, pembrolizumab demonstrated favorable efficacy in patients with recurrent and metastatic HNSCC in a single-arm, phase II KEYNOTE-055 study [12]. Importantly, nivolumab and pembrolizumab have received FDA approval for the treatment of recurrent or metastatic HNSCC patients.

Recently, immune checkpoint inhibitor therapy has been broadly used in the clinical treatment of HNSCC [9,11,12]. Although some patients achieve good clinical benefits, immune resistance and adverse reactions to immunotherapy have been always plaguing clinicians’ decisions [13]. How to judge whether patients can benefit from immunotherapy before receiving ICIs therapy is currently a difficult problem in the clinical treatment of HNSCC patients. The advancement of high-throughput sequencing technology and the improvement and development of bioinformatics provide soil for the decoding of various diseases. For the above reasons, researchers have developed a large number of signatures to predict the immune status and prognostic characteristics of HNSCC patients. Du et al (2022) develop a signature based on metabolically related genes to predict immunotherapy benefit in patients [14]. As the most common post-transcriptional RNA modification in eukaryotic cells, N6-methyladenosine (m6A)-related gene panel has also been used to predict HNSCC prognosis and immunotherapy [15]. However, these models lack large-scale validation and have limited ability to predict the prognosis and immune response of patients in clinical practice.

As we know, T-cell mediated cytotoxicity was the key to tumor immunotherapy. T cell depletion mediated by the tumor microenvironment was an important cause of immune resistance in cancer patients [16]. Improving the sensitivity of tumor cells to T cell killing was an important way to enhance the effect of immunotherapy in patients with HNSCC. Chen et al (2022) found enhanced T cell-mediated killing by injecting an antibody against Interferon gamma into cells. In many human cancers, the expression of PBRM1 and ARID2 enhances the sensitivity of tumor cells to T cell killing, thereby inhibiting tumor progression [17]. Based on the above research, we found that genes associated with the sensitivity of tumor cells to T cell-mediated killing (GSTTK) played an important role in tumor immunotherapy.

In this study, we utilized a panel of GSTTKs to discriminate immune status and prognostic features of HNSCC patients and validated in different cohorts to enhance the robustness of the results. We have developed a novel classification to identify immune-resistant and immune-sensitive populations in HNSCC and assigned corresponding treatment options. The formulation of the GSTTK classification model provides help for the personalized treatment and management of HNSCC patients in clinical practice and greatly improves the clinical benefit of HNSCC patients.

## 2. Methods

### 2.1 Raw data retrieval and preprocessing

A total of 1043 HNSCC samples were procured from the Cancer Genome Atlas (TCGA, https://portal.gdc.cancer.gov/), Gene Expression Omnibus (GEO, http://www.ncbi.nlm.nih.gov/geo/), and ArrayExpress (https://www.ebi.ac.uk/arrayexpress/) databases. All samples were included in five independent datasets, respectively TCGA-HNSC (n = 494), GSE41613 (n = 97), GSE42743 (n = 74), GSE65858 (n = 270), and E_MTAB_8588 (n = 108). The inclusion and exclusion criteria for the research samples were as following: (1) All were primary tumors; (2) had complete clinical information; (3) Preoperative chemotherapy or chemoradiotherapy were not received. In addition, a gene set named GSTTK was established, which consists of genes associated with a satisfactory response to TTK in cancer immunotherapy in the TISIDB database (http://cis.hku.hk/TISIDB/) [18]. All data were z-score transformed prior to analysis.

### 2.2 Integrated multi-omics analysis

We identified critical GSTTK genes through integrative analysis of transcriptome and genomic data. Firstly, to screen for differentially expressed genes (DEGs) in tumor and normal samples, the ‘*edgeR’* R package was utilized. P values <0.05 and abs (log2 fold change) >1is defined as the significance threshold [19]. Secondly, univariate Cox regression analysis was used to screen genes with independent prognosis significance for HNSCC patients (P <0.05). ‘*VennDiagram’* R package was employed to draw a Veen plot displaying the intersection of DEGs and Uni-cox genes. Subsequently, we downloaded the somatic mutation and copy number variation (CNV) data from cBioPortal (https://www.cbioportal.org/) and Firebrowse (http://firebrowse.org/) databases, respectively. We used ‘*maftools’* R package to map the mutation of the intersection of genes [20]. CNV status of each gene was delineated by the Genomic Identification of Significant Targets in Cancer 2.0 (GISTIC2.0) algorithm, which was visualized by ‘*ggplot2*’ and ‘*RCircos*’ R packages. In order to test the ability of GSTTK to discriminate between tumor and normal patients, we analyzed the TCGA-HNSC cohort using the ‘*FactoMineR*’ R package and visualized the results by the ‘fviz_pca_ind’ function.

### 2.3 Identification of GSTTK subtypes

Based on the GSTTK gene obtained in the previous step, we performed an unsupervised clustering analysis in TCGA cohort. Consensus clustering analysis was implemented through the ‘*ConsensusClusterPlus*’ R package [21]. Clustering was completed according to the following parameters: (1) 80% items resampling; (2) The sampling times are 1000; (3) K (the number of clusters) has a minimum of two and a maximum of ten. In order to select the optimal number of subtypes, consensus matrices and cumulative distribution function (CDF) were utilized [22]. After selecting the optimal K value, heat map was used to plot the clinical features and expression characteristics of different subgroups. In addition, the ‘survival’ R package was used to draw the K-M curves and assess prognostic differences in different subgroups.

### 2.4 Identified the eigengene by WGCNA

The important function of weighted gene co-expression network analysis (WGCNA) is to construct scale-free networks using gene expression matrices [23]. Scale-free networks can predict intergenic regulatory relationships using expression correlations among genes. Based on the above reasons, we used the ‘WGCNA’ R package to determine the most relevant gene modules for each subtype, and the module genes were defined as the characteristic genes of the subtype. Here are the detailed calculations: (1) Median absolute deviation (MAD) was calculated and ranked for each gene according to the gene expression matrix, and the top 5000 ranked genes were selected for next calculation. (2) The optimal soft threshold is chosen to ensure that the network has high correlation and connection strength. In this study, we use ‘*powerEstimate*’ parameter to select the best soft threshold and construct scale-free network according to the best soft threshold. (3) We further construct the adjacency matrix and transform the adjacency matrix into a topological overlap matrix (TOM), and 1-TOM matrix describes the similarity between genes and completes hierarchical clustering of genes. (4) To divide genes into different modules, dynamic shear analysis was performed using the ‘*cutreeDynamic*’ function. (5) Since the genes in the modules have strong connectivity, we calculated the correlation between each module and the subtype separately, and the genes in the module with the highest correlation are the eigengene of this subtype.

### 2.5 Validation of clustering results

In order to further assess the prognostic characteristics of different subtypes, we divided four independent cohorts of patients into corresponding subtypes according to the Nearest template prediction (NTP) algorithm. NTP is an efficient method proposed by Hoshida for performing class prediction, which classifies patients based on the expression abundance of eigengene in different cohorts and provides a measure of predictive reliability [24]. Subsequently, we compared prognostic differences between subgroups in all validation cohorts separately, including GSE41613, GSE42743, GSE65858, and E_MTAB_8588. K-M curves were utilized to visualize the results.

### 2.6 Gene set enrichment analysis

Functional enrichment analysis provides convenience to decode the molecular mechanisms underlying disease. In order to explore the enrichment of different pathways in specific gene sets, gene set enrichment analysis (GSEA) was utilized [25]. We performed differential gene analysis between different subgroups and ranked genes according to the log2FoldChange (log2FC) values. This ranked gene list was used as input file GSEA. Subsequently, we used the ‘*clusterProfiler*’ R package to explore specific GO terms and KEGG pathways that were significantly different between different subgroups [26].

### 2.7 Gene set variation analysis

Gene set variation analysis (GSVA) is a particular type of gene set enrichment method that works on single samples and enables pathway-centric analyses of molecular data by performing a conceptually simple but powerful change in the functional unit of analysis, from genes to gene sets. In this study, we used the ‘GSVA’ R package to explore significant differences in function and pathway among patients in different subgroups [27].

### 2.8 Immune cell infiltration and immune checkpoint analysis

Immune status is a key factor in guiding immunotherapy of patients, and the abundance of immune cells and expression level of immune checkpoints are important indicators to measure the immune status of patients. Based on gene expression matrices and specific gene sets for each immune cell, we calculated the abundance of 28 immune cells for each sample using the ssGSEA algorithm [25]. Subsequently, we further compared the expression differences of immune molecules among different subgroups, including co-stimulatory checkpoints and human leukocyte antigen (HLA) family. In addition, we comprehensively assessed the abundance of 27 immune molecules using immunophenoscore (IPS) [28], which mainly came from the TNF and B7-CD28 superfamily.

### 2.9 Evaluation of immunotherapy response

As we know, immunotherapy for patients with high immune status can significantly improve patient survival. In contrast, immunotherapy for patients with low immune status will increase patient costs and cause complications. To explore the extent of response to immunotherapy, we performed SubMap analysis between different subgroups patients. SubMap is an unsupervised clustering approach algorithm and is utilized to evaluate the response rate of patients to PD-1 and CTLA-4 therapy. We also used tumor immune dysfunction and exclusion (TIDE, http://tide.dfci.harvard.edu/) to predict patient response rates to immune checkpoint inhibitors [29]. Additionally, we obtained neoantigen load (NAL) in HNSCC patients from the TCGA website and used boxplots to compare differences in NAL between subgroups.

### 2.10 Mutation landscape

We decoded the mutational landscape of patients in the TCGA-HNSC cohort by the ‘*maftools*’ R package and visualized the genes in the top 20 mutation frequencies using the ‘*oncoplot*’ function. We defined the genes in the top 20 mutation frequencies as key mutated genes (KMGs) and compared the differences in KMGs mutation frequencies between different subgroups. We calculated the total number of mutations in each sample and defined it as tumor mutation burden (TMB), which includes coding, indel, and base substitution mutations, etc. Subsequently, we used the boxplot to describe the differences in mutation load between different subgroups, including TMB, insertion deletion (INDEL), and single nucleotide variations (SNV). Additionally, the ‘*somaticInteractions*’ function was used to explore co-mutation and exclusion phenomena among KMGs.

### 2.11 Copy number variation

To explore the CNV landscape in HNSCC patients, we contrasted the extent of CNV in patients with different clinical features and visualized the results by ggplot2, which includes fraction of genome altered (FGA), fraction of genome gained (FGG), and fraction of genome lose (FGL). Subsequently, we calculated the copy number gistic score for patients using the GISTIC 2.0 algorithm.

### 2.12 Assessment of clinical treatment benefit

In order to assess the clinical treatment benefit of HNSCC patients, we compared sensitivity to chemotherapeutic agents and immunotherapy in different subgroups of patients. Firstly, we collected the gene expression and drug sensitivity information in HNSCC cell lines from GDSC and CTRP databases. The NTP algorithm was used to divide HNSCC cell lines into different subgroups. We also compared the sensitivity of different subgroups of cell lines to first-line chemotherapeutic agents (including 5-fluorouracil, dasatinib, platinum, etc). Whereafter, we compared the response rates of different subtypes to immunotherapy in multiple immunotherapy cohorts, including GSE35640, GSE173839, IMvigor210, GSE91061, GSE135222, and VanAllen cohort.

### 2.13 Statistical analysis

In the R software (version 4.1.2), we completed all data processing, calculation, analysis, and visualization. T-test and Wilcoxon rank-sum test were utilized to compare the continuous variables and the comparisons among categorical variables were calculated by the chi-square test. It was considered significant when the P value was less than 0.05 in all two-sided tests.

## 3. Results

### 3.1 Identification of GSTTKs associated with HNSCC progression

**Fig 1:**
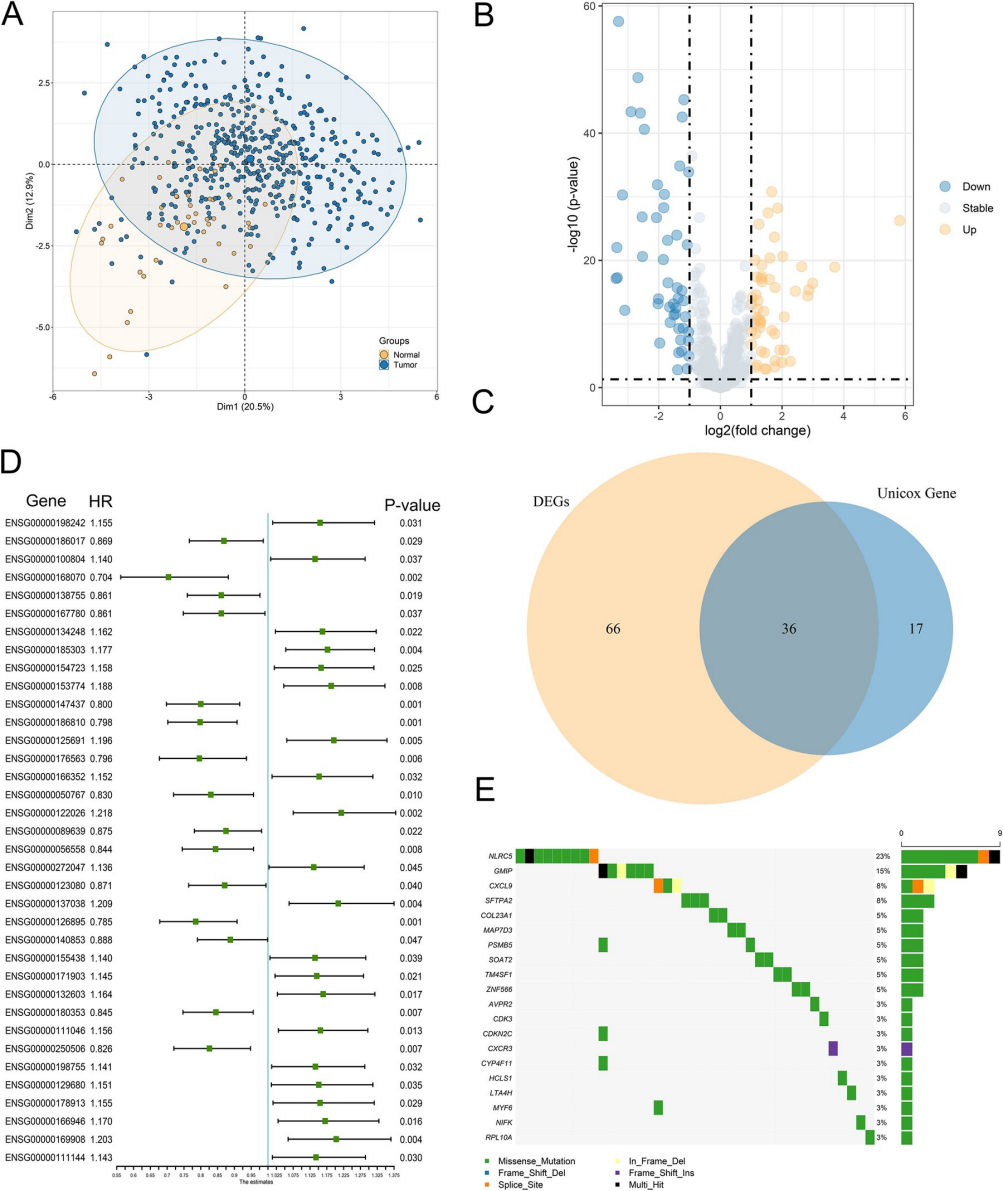
Identification of GSTTKs and detection of mutations in HNSCC. (**A**) Principal component analysis used GSTTK to separate tumor and normal samples. (**B**) Volcano plot displayed 102 differential expression GSTTK between tumor and normal samples in the TCGA-HNSC cohort. (**C**) Venn diagram shows 36 GSTTKs exhibiting both differential expression and prognostic value in HNSCC. (**D**) Univariate Cox regression analysis of 36 GSTTKs associated with clinical prognosis in HNSCC. (**E**) Waterfall plot demonstrated the mutational landscape of 20 GSTTKs with mutation frequencies greater than zero.

**Fig 2:**
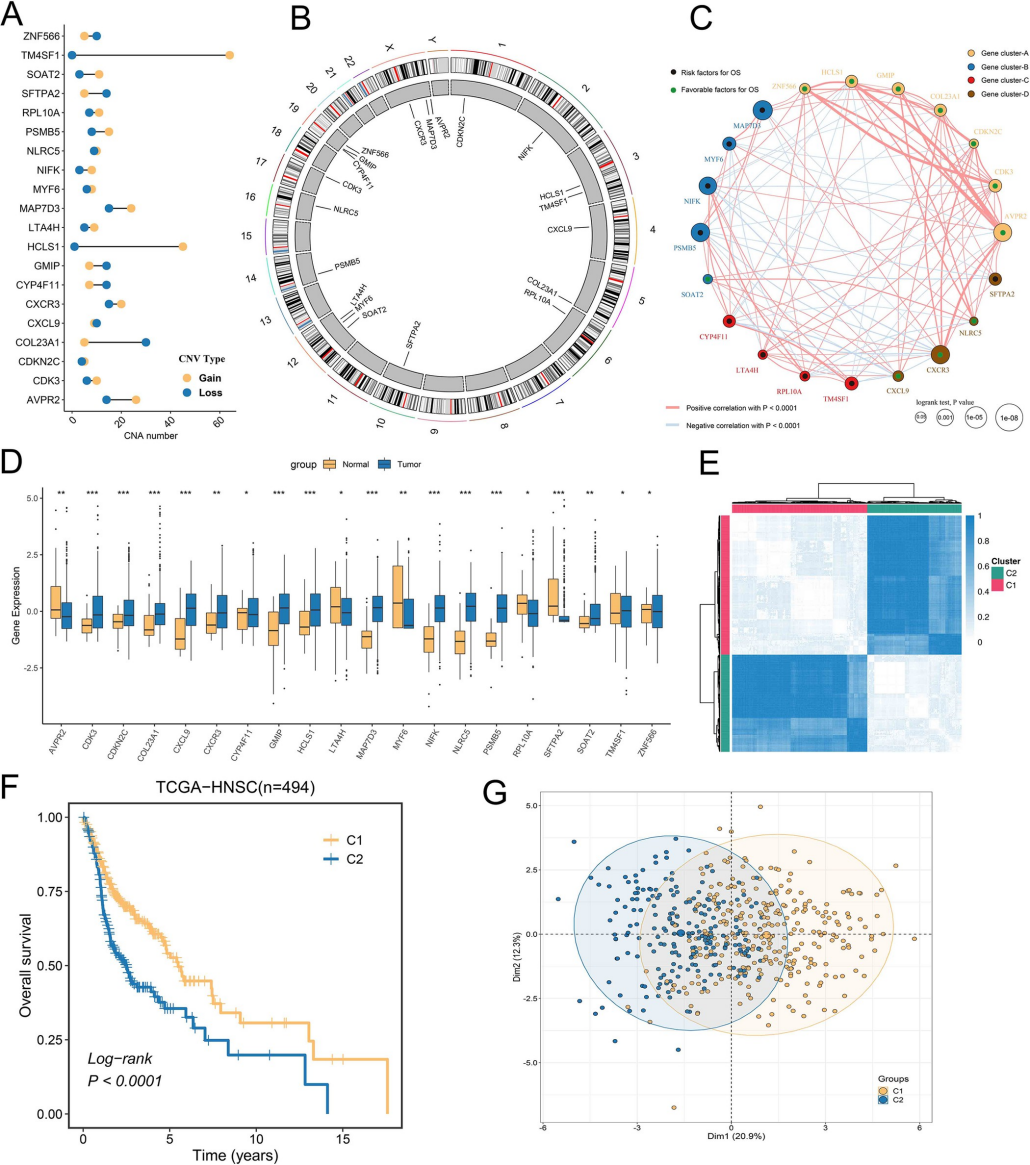
Patterns of TTK and their prognostic value in HNSCC. (**A**) Copy number variation (CNV) in 20 GSTTKs in HNSCC. (**B**) The location of CNV alteration of 20 GSTTKs on 23 chromosomes using TCGA-HNSC cohort. (**C**) Interactions among 20 GSTTKs in HNSCC. Gene cluster A, blue; gene cluster B, red; gene cluster C, brown; gene cluster D, orange. The size of each cell represents survival impact of GSTTK, calculation used the formula log10 (log-rank test P values indicated). Favorable factors for overall survival are indicated in green, and risk factors indicated in black. The lines connecting GSTTK represent gene interactions. The thickness of the line represents the strength of correlation estimated by Spearman correlation analysis. Positive correlation is indicated in red and negative correlation in blue. (**D**) The boxplot showed the expression levels of 20 GSTTKs in tumors and normal tissues. (**E**) Two patterns of TTK were identified by consistent clustering. (**F**) Survival analysis indicated that patients assigned to the two clusters had significantly different survival outcomes in TCGA-LIHC. (**G**) Principal component analysis used GSTTK to separate C1 and C2 samples.

The analysis pipeline of our study was displayed in S1 Fig. Multi-omics analysis of GSTTKs using multi-omics data from the TCGA-HNSC cohort. PCA displayed that GSTTKs can well separate HNSCC from normal samples (Fig 1A), indicating that GSTTKs played an important role in the occurrence and development of HNSCC. As shown in the volcano plot (Fig 1B), we identified a total of 102 significantly dysregulated GSTTKs by differential analysis of tumor and normal sample, including 53 up-regulated and 49 down-regulated. Univariate Cox regression analysis showed that 53 of 593 GSTTKs were significantly associated with the prognosis of HNSCC. A total of 36 GSTTKs with significant differences and prognostic values were obtained by intersecting the two parts of genes by Veen plot (Fig 1C). Results of univariate Cox analysis of 36 GSTTKs were presented using forest plots (Fig 1D). We found that 36 GSTTKs included 15 protective factors (Hazard Ratio [HR] <1) and 21 risk factors (HR >1). We further analyzed somatic mutation data from the TCGA-HNSC cohort and delineated the mutational landscape of 36 GSTTKs by waterfall plots (Fig 1E). Remarkably, only 20 GSTTKs were mutated and had a frequency greater than 1%, including NLRC5, GMIP, CXCL9, SFTPA2, COL23A1, MAP7D3, PSMB5, SOAT2, etc. They play an important role in the development and progression of HNSCC. Additionally, we explored the presence of prevalent CNV variants in these 20 GSTTKs (Fig 2A). TM4SF1, HCLS1, AVPR2, and MAP7D3 showed significant copy number gain, and copy number loss was more significant in COL23A1, SFTPA2, GMIP, and CYP4F11. As displayed in Fig 2B, we delineated the localization of 20 GSTTKs on chromosomes in detail. Subsequently, we comprehensively explored the interaction relationship and prognostic value of 20 GSTTKs using clinical information and transcriptomic characterization of the TCGA-HNSC cohort and divided them into four patterns (Fig 2B). Finally, we further compared the expression differences of 20 GSTTKs in tumor and normal samples by boxplot (Fig 2C).

### 3.2 Identification of two HNSCCC subtypes with prognostic significance

**Fig 3:**
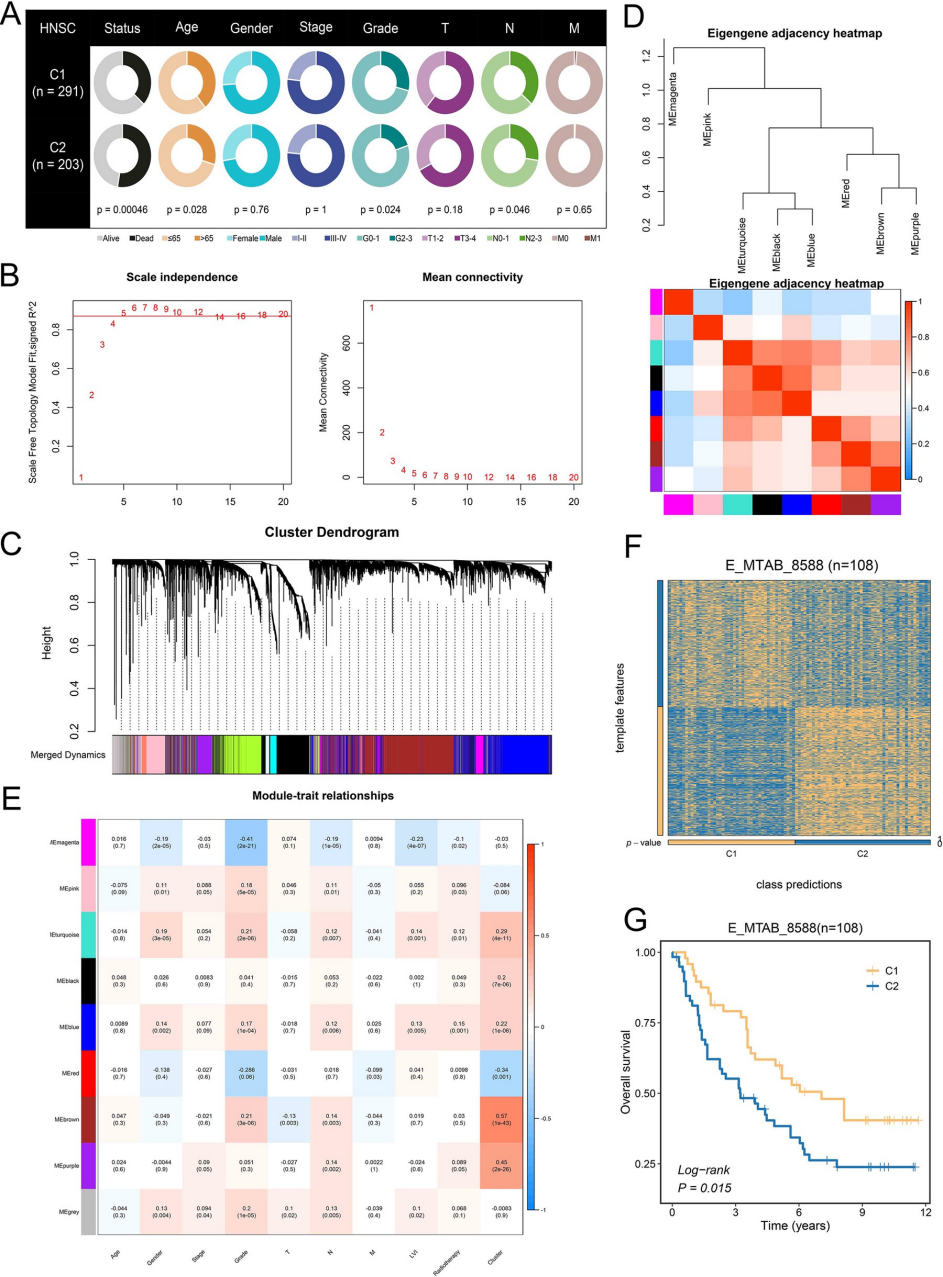
Identification of characteristic genes in different subgroups of patients by WGCNA. (**A**) Clinical features of patients in the C1 and C2 subgroups. (**B**) Analysis of network topology for different soft-threshold power. The left panel shows the impact of soft-threshold power on the scale-free topology fit index; the right panel displays the impact of soft-threshold power on the mean connectivity. (**C**) Gene clustering tree (dendrogram) obtained by hierarchical clustering of adjacency-based dissimilarity. (**D**) Heat map of the eigengene adjacency. The color bars on the left and below indicate the modules for each row or column. (**E**) Heatmap between gene modules and clinical characteristics. (**F**) Heatmap of the expression level of the template feature between two subgroups in the E_MTAB_8588 cohort. (**G**) Kaplan-Meier curves of OS between the two subgroups in the E_MTAB_8588 cohort.

We extracted the expression matrix for 20 GSTTKs from 494 patients in the TCGA-HNSC cohort. K = 2 was identified as the stable and optimal number of clusters by the ‘*ConsensusCluster*’ algorithm (S2 Fig). As shown in Fig 2E, patients were divided into C1 (n = 291) and C2 (n = 203) subtypes. Additionally, the gene expression heatmap of 20 GSTTKs displayed that PSMB5, MYF6, MAP7D3, NIFK, et al. were highly expressed in C1 subtype, and CXCR3, ZNF566, SFTPA2, HCLS1, NLRC5, et al. was more expressed in C2 subtype (S3 Fig). Notably, the KM curve revealed a significant difference in prognosis between the two subgroups of patients. Compared with C1 subtype, patients in C2 subtype displayed a dismal prognosis (Fig 2F). Additionally, we utilized PCA algorithm to assess the differences between the GSTTK gene profiles of the two subgroups, and we found that there were significant expression differences between the two GSTTK patterns (Fig 2G). As illustrated in Fig 3A, we further evaluation of clinical features and found that there were significant differences between the two subgroups in terms of survival status, age, Grade, and N stage.

### 3.3 Brown and red model have high correlation with subgroups

WGCNA can identify subtle interactions in the gene network and classify genes into co-expression modules based on their associated roles among networks. Based on the above characteristics, we utilized WGCNA to explore the characteristic gene module of the two subgroups. We selected the genes with the top 5000 MAD values in the TCGA-HNSC cohort to construct a co-expression network. As illustrated in Fig 3B, five was defined as the optimal soft threshold. As this time, the network has high correlation and connectivity. Subsequently, we constructed the co-expression module through dynamic shear tree algorithm (Fig 3C). The 5000 genes were divided into nine gene modules and number of genes in different modules was display in the S1 Table. Eigengene adjacency heatmap displays the correlation between modules (Fig 3D). Subsequently, we calculated the correlation of each module with subgroups and found that brown model was most associated with C1 subtype (|r| = 0.57, P <0.001) and red model was most associated with C2 subtype (|r| = 0.34, P = 0.001, Fig 3E). Therefore, gene in the brown module was identified as characteristic genes of C1 subtype, including 911 genes. Likewise, the characteristic genes of C2 subtype were assumed by the red module gene, including 802 genes.

### 3.4 Validation of TTK subtypes in four independent cohorts

**Fig 4:**
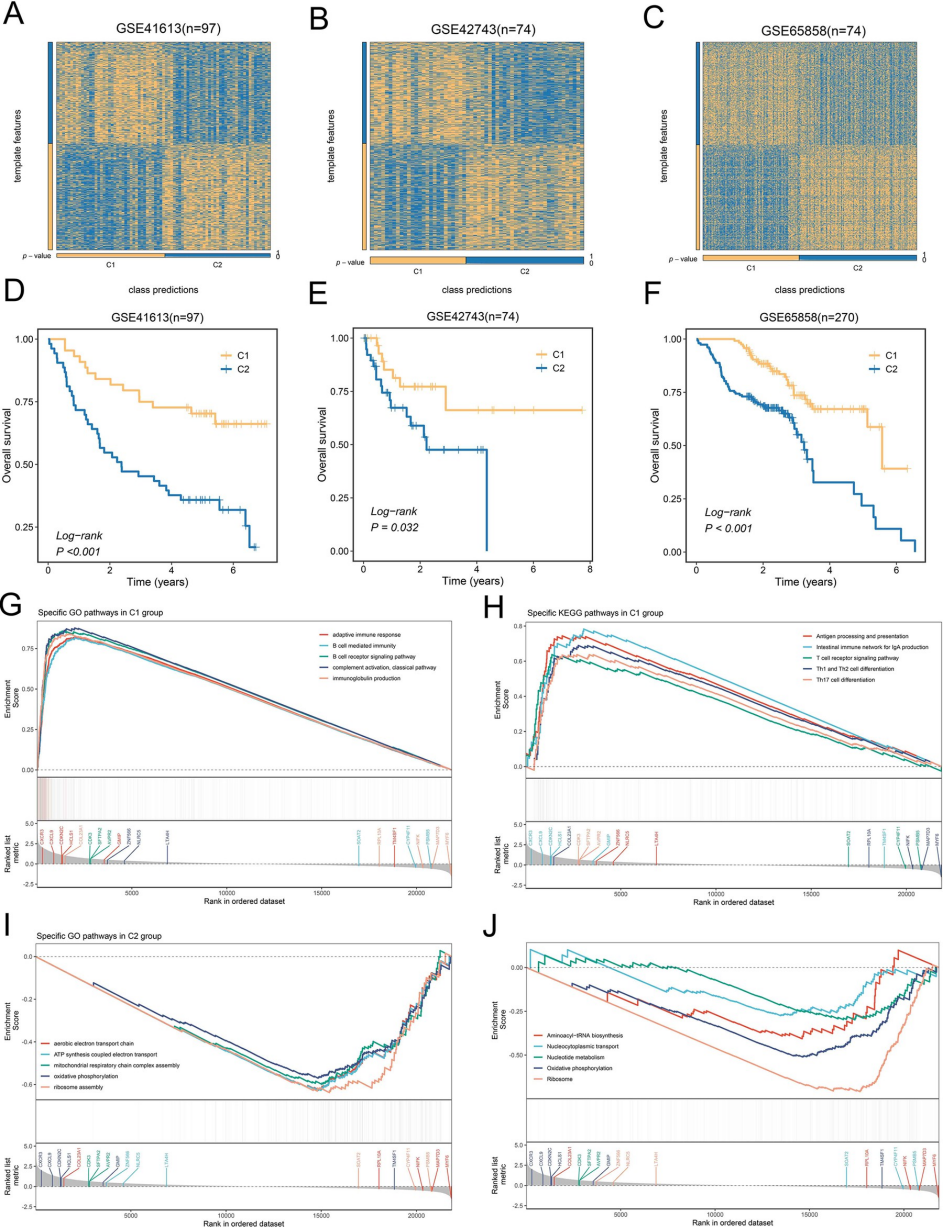
The evaluation of TTK patterns and explored the potential mechanisms. (**A-C**) Heatmap of the expression level of the template feature between two subgroups in the GSE41613 (**A**), GSE42743(**B**), and GSE65858 (**C**). (**D-F**) Kaplan-Meier curves of OS between the two subgroups in the GSE41613 (**D**), GSE42743(**E**), and GSE65858 (**F**). (**G-J**) The top five GO terms and KEGG pathways in the two subgroups.

Although TTK subtypes displayed independent prognostic significance in the TCGA-HNSC cohort, the robustness of this classification requires further validation. Therefore, we divided HNSCC patients into TTK subtypes by NTP algorithm in four independent cohorts. Heatmap displayed favorable classification of all cohorts, including E_MTAB_8588 (Fig 3F), GSE41613 (Fig 4A), GSE42743(Fig 4B), GSE65858 (Fig 4C). Notably, KM curve displayed significant difference in prognosis between C1 and C2 subtypes in all cohort, including E_MTAB_8588 (P = 0.015, Fig 3G), GSE41613 (P <0.001, Fig 4D), GSE42743(P = 0.032, Fig 4E), GSE65858 (P <0.001, Fig 4F).

### 3.5 C2 subgroup exhibited robust metabolism-related features

We utilized the GSEA algorithm to reveal the enriched status of C1 and C2 subgroups in GO functions and KEGG pathways. As shown in Fig 4G and 4H, C1 subgroup patients were mainly concentrated in immune-related functions and pathways, including adaptive immune response, B cell mediated immunity, immunoglobulin production, antigen processing and presentation, T cell receptor signaling pathway, Th1 and Th2 cell differentiation, and Th17 cell differentiation. Likewise, C2 subgroup patients were mainly focused on metabolism-related functions and pathways, such as aerobic electron transport chain, ATP synthesis coupled electron transport, mitochondrial respiratory chain complex assembly, aminoacyl-tRNA biosynthesis, nucleotide metabolism, and oxidative phosphorylation (Fig 4I and 4J). Additionally, the GSVA algorithm also was used to explore significant functional and pathway differences in the C1 and C2 subgroups (S4 Fig). C1 subgroup patients were significantly enriched in allograft rejection, IL2 stat5 signaling, and notch signaling, et al. In contrast, C2 subgroup patients were significantly enriched in myogenesis, pancreas beta cells, p53 pathway, et al.

### 3.6 Patients with C1 subgroup showed higher abundance of immune infiltrates

**Fig 5:**
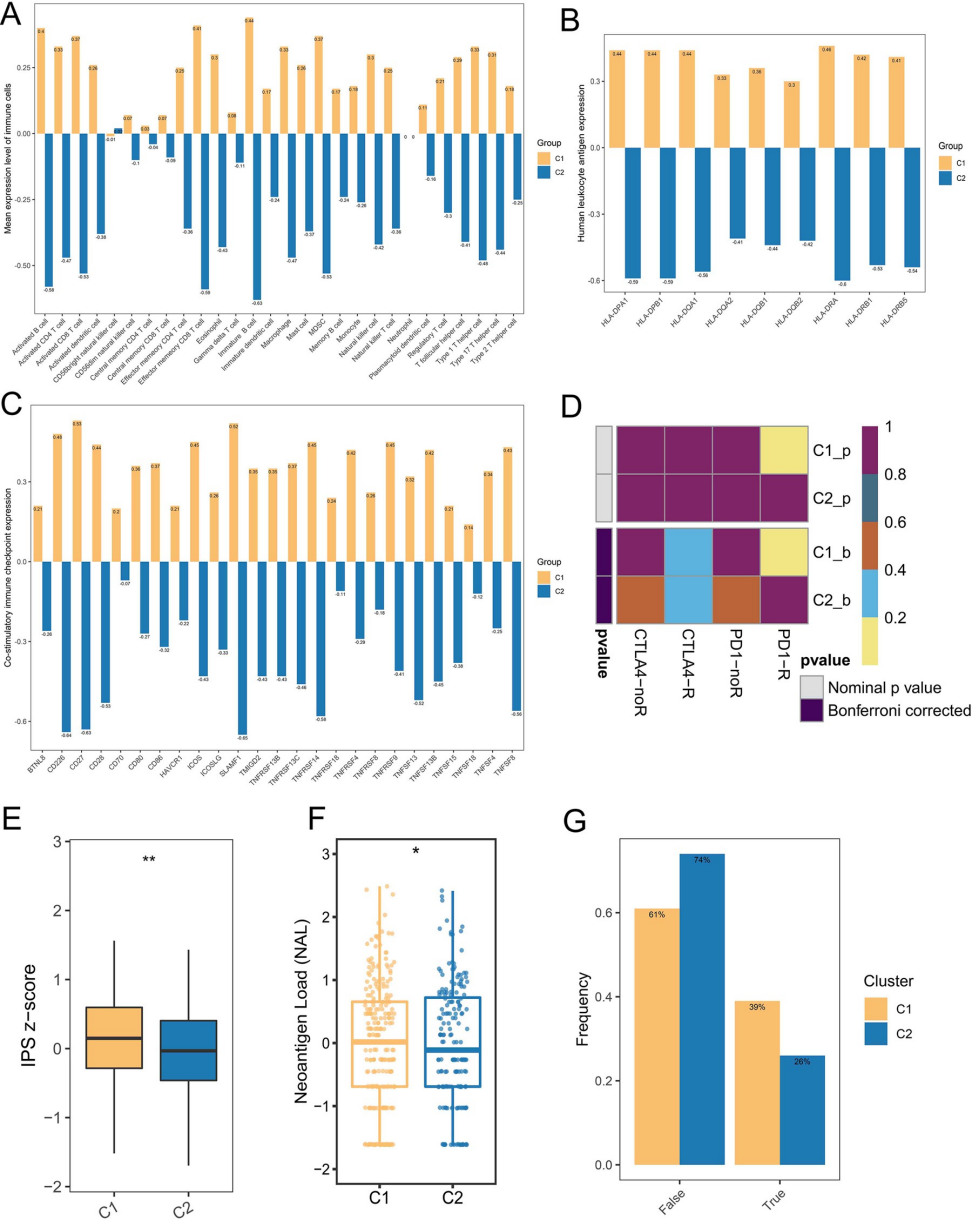
Immune assessment between the two subgroups. (**A**) The abundance of 28 immune cells. (**B**) Comparison of HLA family molecule between the two subgroups. (**C**) Comparison of immune co-stimulatory between the two subgroups. (**D**) SubMap algorithm evaluated the expression similarity between the two phenotypes and the patients with different immunotherapy responses. (**E-G**) Comparison of IPS (**E**), NAL (**F**), and TIDE (**G**) score between the two subgroups.

Based on the results of GSEA and GSVA, we found that patients with C1 subgroup demonstrated more robust immune characteristics. In order to further assess the abundance of immune infiltrates in different subgroups, ssGSEA algorithm was utilized to calculate the expression of 28 immune cells. As showcased in Fig 5A, by comparing the expression levels of 28 types of immune cells, we found that expression levels of immune cells in C1 subgroup patients was significantly higher than that in C2 subgroup patients. Likewise, compared with C2 subgroup, immune molecules were higher in C1 subgroup patients, including HLA molecules family (HLA-DPA1, HLA-DPB1, HLA-DQA1, HLA-DQA2, et al. Fig 5B) and immune co-stimulatory molecules (BTNL8, CD226, CD27, CD28, TNFSF8, TNFSF4, et al. Fig 5C). Subsequently, SubMap algorithm was utilized to evaluate the response rates to PD-1 and CTLA4 in C1 and C2 subgroups patients (Fig 5D). Notably, patients with C1 subgroup were more sensitive to PD-1 therapy than C2 subgroup patients. However, there was no significant difference in response rates to CTLA4 between the two subgroups (Fig 5D). Additionally, compared with C2 subgroup, patients in C1 subgroup had significantly higher IPS score (Fig 5E) and neoantigen burden (Fig 5F). As illustrated in Fig 5G, TIDE algorithm analysis results suggest that patients with C1 subgroup had a higher true response rate to immunotherapy.

### 3.7 Significant mutational burden was exhibited in C1 subgroup patients

**Fig 6:**
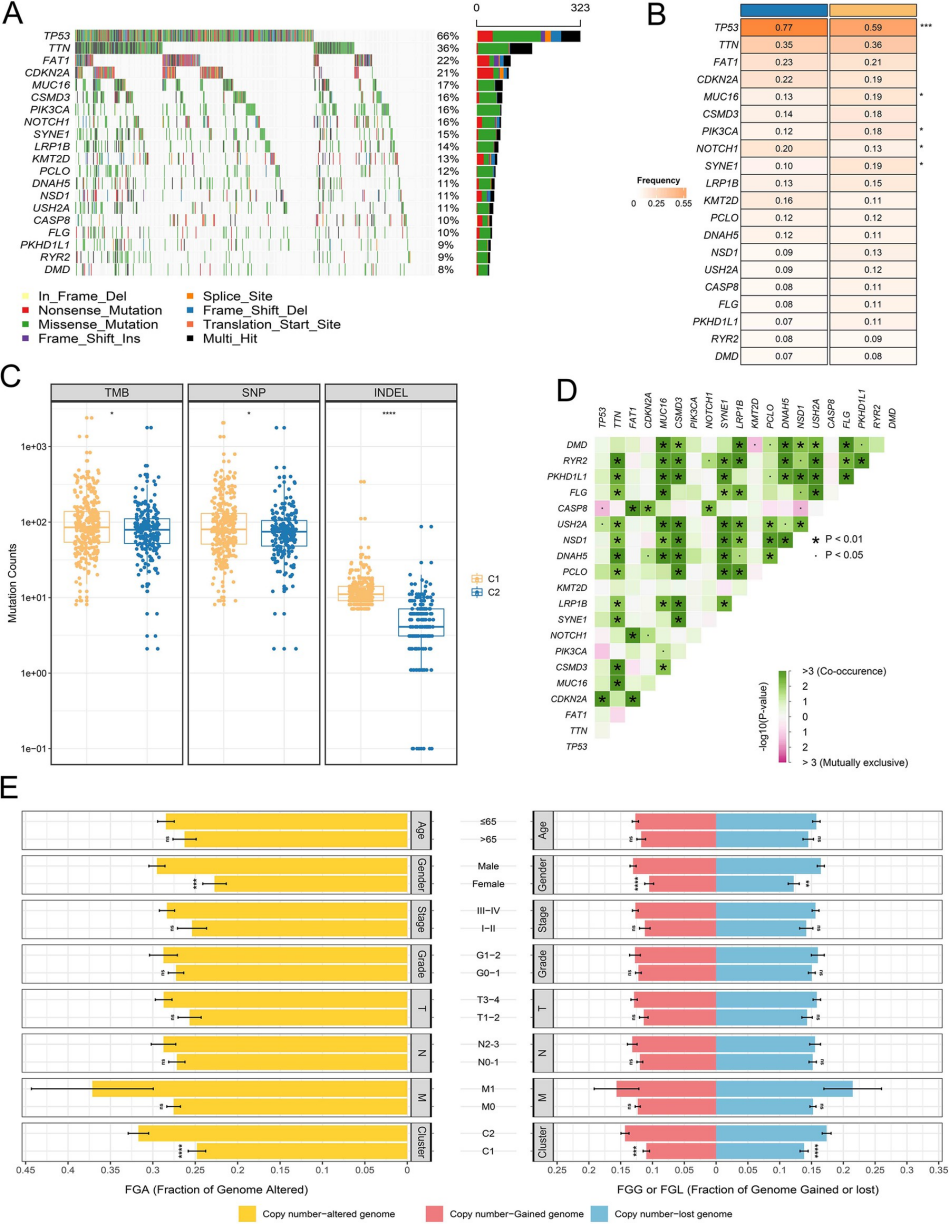
Genomic variation analysis. (**A-B**) Mutational landscape (**A**) and frequency (**B**) of the top 20 KMGs between two subgroups. (**C**) Comparison of TMB, SNP, and INDEL between the two subgroups. (**D**) Co-mutation and co-exclusion of the 20 GSTTKS. (**E**) Comparison of CNV between different subgroups.

Waterfall plot displayed the mutation landscape of the top 20 genes with mutation frequency in the TCGA-HNSC cohort, including TP53 (66%), TTN (36%), FAT1 (22%), CDKN2A (21%), MUC16 (17%), CSMD3 (16%), et al. (Fig 6A). The types of mutation mainly focus on missense mutation, nonsense mutation, and multi-hit (Fig 6A). Further comparison of the mutation frequencies of KMGs between the two subgroups found that TP53 and NOTCH1 had higher frequencies in the C2 subgroup, MUC16, PIK3CA, and SYNE1 had higher frequencies in the C1 subgroup (Fig 6B). Subsequently, we found significant mutational burden was exhibited in C1 subgroup patients through boxplots, including TMB, SNP, and INDEL (Fig 6C). Notably, KMGs demonstrated strong co-mutation phenomenon, including TTN-RYR2, MUC16-DMD, LRP1B-FLG, and NSD1-USH2A et al. (Fig 6D).

### 3.8 Genomic variants appear more frequently in patients with C2 subgroup

**Fig 7:**
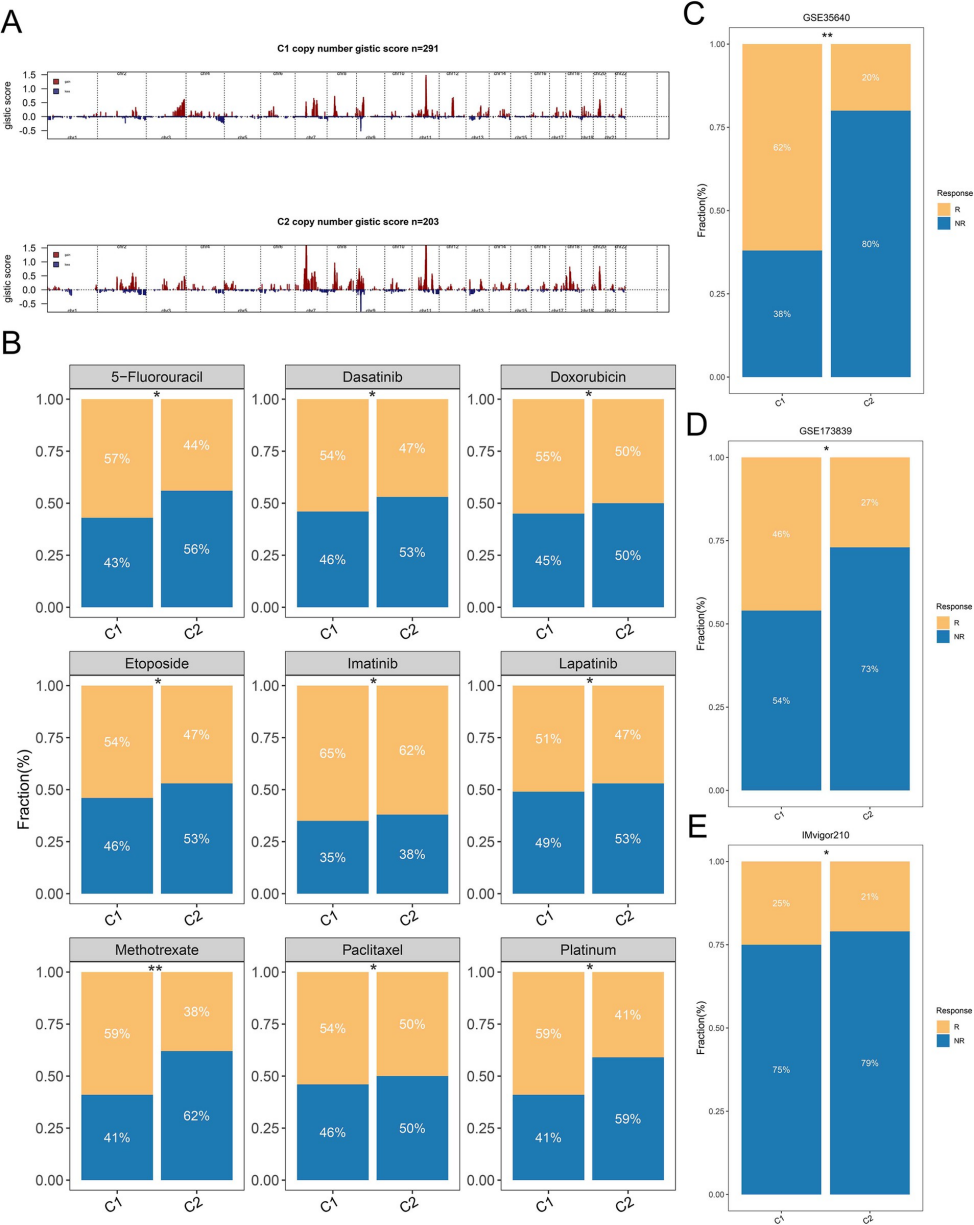
Evaluation of the benefits of chemotherapy and immunotherapy. (**A**) Comparison of gistic score between different subgroups. (**B**) Sensitivity of HNSCC cell lines to nine chemotherapy drugs in different subgroups. (**C-E**) Evaluation of immunotherapy effect between two subgroups in the GSE35640 (**C**), GSE173839 (**D**), and IMvigor210 (**E**).

We further calculated the frequency of CNV in HNSCC patients and compared the CNV in different clinical characteristics (Fig 6E). Notably, C2 subtype patients have significantly higher CNV frequencies than C1 subtype patients, including FGA, FGG, and FGL. Compared with the female, male patients have a higher frequency of genomic variants. Additionally, we found that genomic variants in HNSCC patients were mainly concentrated in chromosomes three and eight (S5 Fig). Patients with C2 subgroup were found to have higher copy number gistic score, which was calculated by GISTIC 2.0 algorithm (Fig 7A).

### 3.9 C1 subgroup patients were more sensitive to immunotherapy and chemotherapy

We collected the gene expression and drug sensitivity data of 71 HNSCC cells from CTRP and GDSC databases. As shown in Fig 7B, patients with C1 subgroup were more sensitive to multiple first-line chemotherapy agents, including 5-fluorouracil, dasatinib, platinum, doxorubicin, etoposide, imatinib, lapatinib, methotrexate, and paclitaxel. In addition, we collected six immunotherapy cohorts from a variety of cancers. Patients with subgroup C1 had significantly higher response rates to immunotherapy than those with subgroup C2 in all cohorts, such as GSE35640 (Fig 7C), GSE173839 (Fig 7D), IMvigor210 (Fig 7E), GSE91061 (S6A Fig), GSE135222 (S6B Fig), and VanAllen (S6C Fig).

## 4. Discussion

Despite the tremendous progress made in medicine in the past 20 years, how to prolong the survival of cancer patients is still a great challenge for all medical researchers. Compared with other solid tumors, HNSCC has higher degree of malignancy and risk of recurrence and metastasis [2,10]. The advent of immunotherapy drugs is a breakthrough in the treatment of HNSCC, including nivolumab and pembrolizumab, etc. Nevertheless, the clinical effect of HNSCC patients receiving immunotherapy was not ideal. Immune resistance and immune complications have always plagued the decision-making of clinicians [9–12]. T cell-mediated immunotoxicity is critical for cancer immunotherapy [17]. Improving the sensitivity of tumor cells to T cell killing is an important modality to relieve immune resistance in patients. Therefore, we grouped patients based on GSTTK to identify immunotherapy-sensitive populations and developed rational treatment regimens for different subgroups of patients.

In this study, we successfully screened 20 GSTTK important for the development and progression of HNSCC based on transcriptome expression matrices and somatic mutation information from the TCGA cohort. We constructed TTK patterns for the first time in HNSCC through the 20 GSTTK, which included C1 and C2 subgroups. The significant difference in prognostic value between different subgroups is the prerequisite for favorable classification performance [30]. In the TCGA-HNSC cohort, the survival curves of the TTK patterns were significantly different. Compared with C1 subgroup patients, C2 subgroup patients displayed a more dismal prognosis. Applicability to different cohorts is the basis for the transition of classification models from the laboratory into clinical practice [31]. The lack of large-scale cohort validation of existing classification models results in low performance in clinical practice. Notably, the same subgroups patients demonstrated similar prognostic characteristics in four independent validation cohorts, which further indicates that our developed TTK patterns have strong clinical generalization and applicability.

The prognostic differences among different subgroups often suggest that they have different enriched functional characteristics and molecular mechanisms [32]. GSEA enrichment analysis revealed that C1 subgroup patients displayed a rich immune landscape, and C2 subgroup patients were enriched in metabolism-related functions and pathways. As we know, abundant immune infiltration underlies the immune response in cancer patients. Zhu et al. found that patients with high immune infiltration status had longer overall survival in HNSCC [33]. In our research, we found that the C1 subtype has higher immune status through a variety of methods, including ssGSEA, immune checkpoint, SubMap, TIDE, IPS, and NAL. In addition, the proliferation and migration of tumor cells was important biological behavior of tumor progression, which was closely related to a variety of metabolic pathways, including arginine metabolism, choline metabolism, and ketone-body metabolism [34–36]. In our study, hypermetabolic patients also exhibited dismal prognostic characteristics, which further demonstrated the accuracy and plausibility of our classification.

Complex genetic and genomic alterations may account for the heterogeneity of HNSCC [37]. This high intratumor and intertumoral heterogeneity is an important reason for the failure of clinical chemoradiotherapy [37,38]. Waterfall plot depicted the mutational landscape of the top 20 genes of mutation frequency in HNSCC patients, including TTN, TP53, FAT1, CDKN2A, MUC16, CSMD3, etc. Further comparison of the mutation frequency of KMGs between the two subgroups found that there was significant difference in TP53, MUC16, PIK3CA, NOTCH1, and SYNE1. As a frequently mutated gene in HNSCC, TP53 mutation was significantly associated with poor prognosis in HNSCC patients [39]. Zhang et al. found that MUC16 mutations contribute to the improvement of patient prognosis by promoting the response of solid tumors to immunotherapy [40]. Furthermore, a sequencing study of 51 Chinese HNSCC patients found that NOTHC1 mutation was significantly associated with poor prognosis in HNSCC patients and promoted tumorigenesis and progression [41]. However, the roles of PIK3CA and SYNE1 mutations in HNSCC still require further studies to discover. In addition, we found significant co-mutation among KMGs, which suggested that a strong association of KMGs mutations in HNSCC patients. A comprehensive comparison of mutation burden in patients with different subtypes revealed that TMB, SNP, and INDEL were significantly higher in subtype C1 than those in subtype C2 patients. As we know, as a predictive biomarker of immune checkpoint inhibitors, TMB improves the immune response of tumor patients by attracting effector cells of the immune system by affecting the infiltration signal of immune cells [42]. Patients in C1 subgroup were also found to have high TMB and abundance of immune cell infiltration in our study, which further demonstrated the accuracy of our research.

Major genetic changes contributing to tumor development include point mutations and copy number variations. With the development of deep sequencing technology and genomics, scientists have utilized gene copy number variation in a variety of tumors to predict the prognostic value of tumor patients. Heish et al. explored the relationship between CNV and pancreatic cancer prognosis found that SMAD4 deletion will lead to poor patient prognosis [43]. Previous evidence suggests that even under the influence of environmental factors and clinical characteristics, HNSCC can be reasonably distinguished into different subtypes by important variants at the molecular level. A case-control study assessed the impact of DNA copy number on the risk of survival in HNSCC patients and found that increased CNV was significantly associated with poor patient prognosis [44]. Our study similarly found that patients with higher CNV (C2 subgroup) had more dismal prognosis, which was consistent with previous findings.

Reasonable stratification of patients is the premise to promote clinical personalized treatment, and identifying sensitive chemotherapeutic and immunotherapeutic drugs for different subgroups of patients is the key to improving the clinical benefit of HNSCC patients. Based on the CTRP and GDSC databases, we identified nine sensitive first-line chemotherapeutic agents (platinum, doxorubicin, etoposide, imatinib, lapatinib, etc.) for patients with C1 subgroup. As an important component of the standard of care for advanced HNSCC, platinum-based chemotherapy can significantly improve the overall survival of patients [45]. Fluorouracil has also been used to improve the prognostic value of HNSCC patients [46]. Several other chemotherapeutic agents were widely used in a variety of solid tumors, however their apply in HNSCC lacks corresponding researches [31,47]. Our study stratified patients rationally and developed efficient treatment plans for different groups of patients. Overall, the establishment of GSTTK provides guidance and help for the personalized management and treatment of HNSCC patients in the clinic.

Our study is the first to identify TTK patterns in HNSCC patients by consensus clustering and has the following advantages: (1) Two subgroups of patients had significant prognostic differences and were validated in multiple cohorts. (2) Important GSTTK in HNSCC was identified and immune profiles of patients with different subgroups were delineated. (3) Identify sensitive drugs in different subgroups of patients and drive the process of personalized treatment of HNSCC in the clinic. Although our study needs to be further validated by large-scale clinical trials, TTK patterns have shown excellent results and can provide help and guidance for personalized management and treatment of patients.

## 5. Conclusion

In summary, we identified 20 GSTTK that played critical roles in the development and progression of HNSCC and successfully decoded two TTK patterns through consensus clustering. Notably, the two TTK patterns showed significant prognostic differences in 1063 samples from five centers. Subsequently, we further explored the immune landscape, function characteristics, genome variation, and pharmacological landscape of TTK patterns, which have significant value in predicting the benefit of chemotherapy and immunotherapy. Overall, the establishment of GSTTK provides guidance and assistance to clinicians in the personalized management and treatment of HNSCC patients.

## Supporting information

S1 Fig Flowchart of analysis procedure. (DOCX)

S2 Fig CDF curve of Consistent clustering. (DOCX)

S3 Fig The expression of 20 GSTTKs was displayed by heatmap. (DOCX)

S4 Fig GSVA enrichment analysis. (DOCX)

S5 Fig Copy number percentage of HNSCC patients in TCGA-HNSC cohort. (DOCX)

S6 Fig Evaluation of immunotherapy effect between two subgroups in the GSE91061(A), GSE135222 (B), and VanAllen (C).
(DOCX)

S1 Table Number of genes in different modules. (XLSX)

S1 File (ZIP)
