# Supplementary figures and images for: T cell-mediated tumor killing patterns in head and neck squamous cell carcinoma identify novel molecular subtypes, with prognosis and therapeutic implications
Source: PLoS One. 2023 May 16;18(5):e0285832. doi: 10.1371/journal.pone.0285832 (PMC10187926; doi:10.1371/journal.pone.0285832)

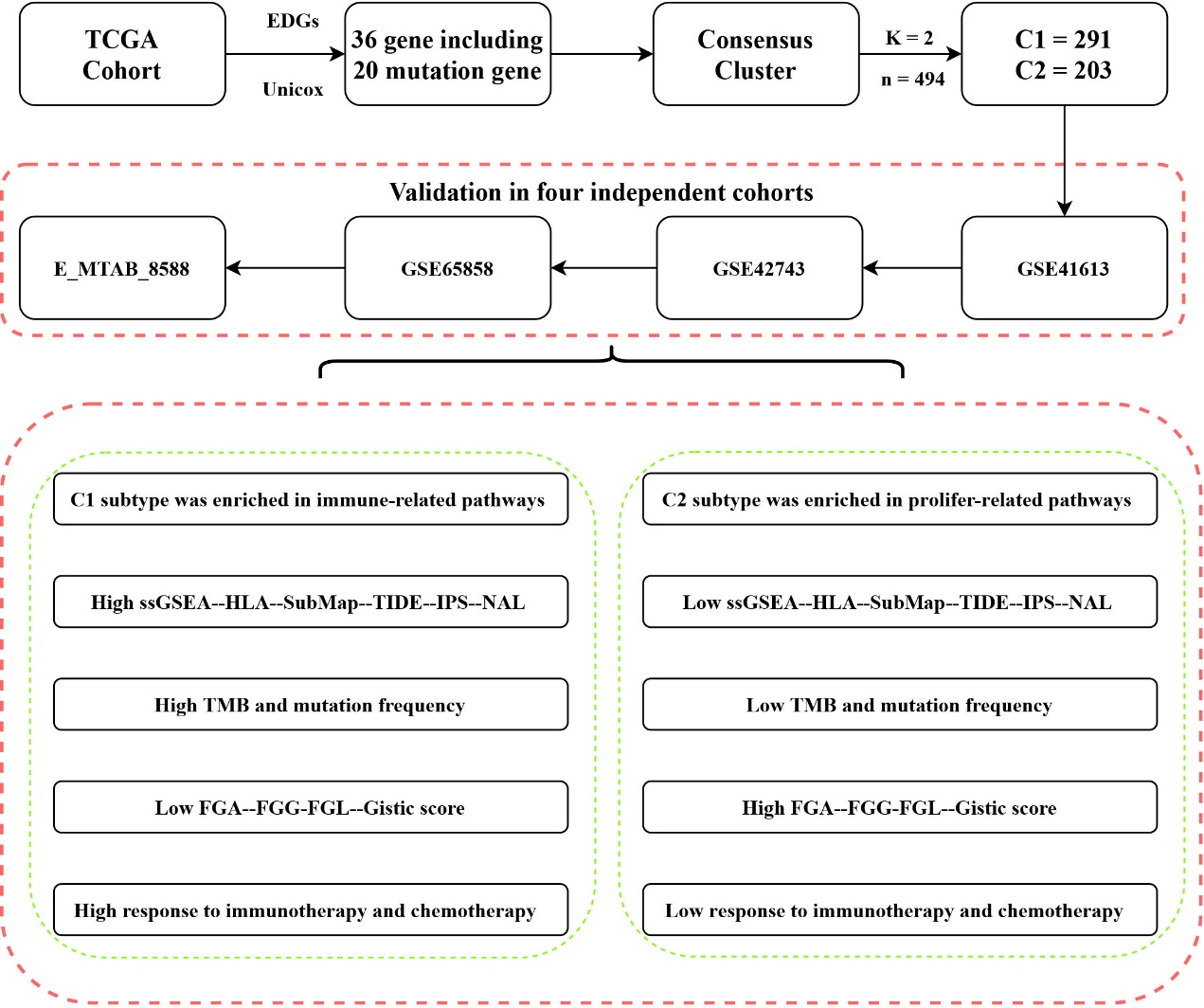


**Figure 1: Flowchart of analysis procedure.**

Supplement: S1 Fig — (DOCX) [file pone.0285832.s001.docx]

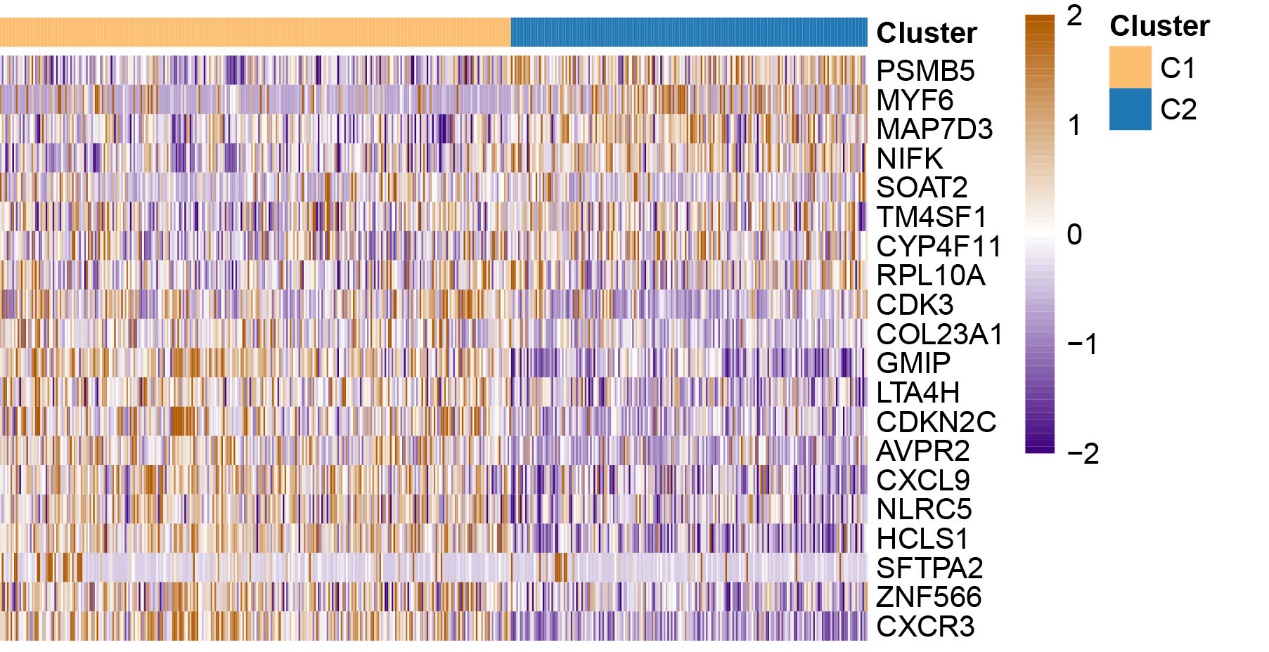


**Figure S3**. **The expression of 20 GSTTKs was displayed by heatmap.**

Supplement: S3 Fig — (DOCX) [file pone.0285832.s003.docx]

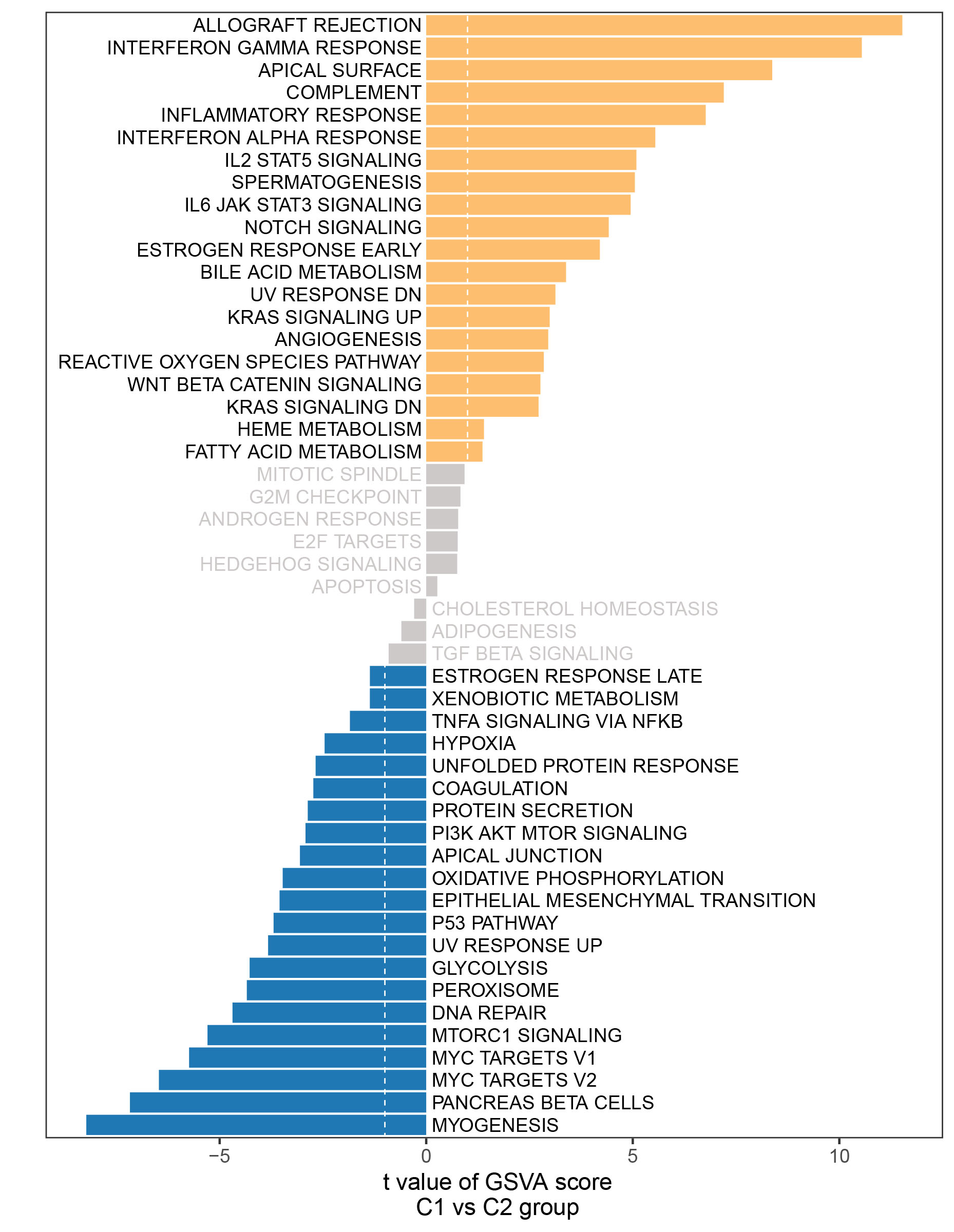


**Figure S4. GSVA enrichment analysis.**

Supplement: S4 Fig — (DOCX) [file pone.0285832.s004.docx]

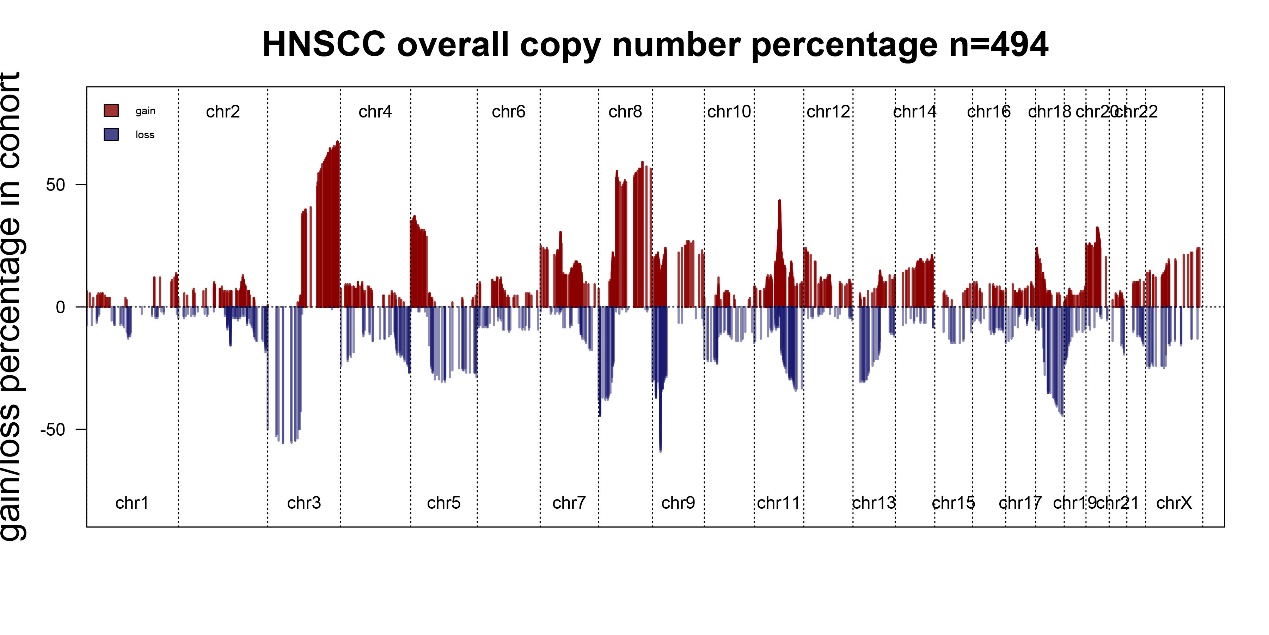


**Figure S5. Copy number percentage of HNSCC patients in TCGA-HNSC cohort.**

Supplement: S5 Fig — (DOCX) [file pone.0285832.s005.docx]
