# Supplementary material for: T cell-mediated tumor killing patterns in head and neck squamous cell carcinoma identify novel molecular subtypes, with prognosis and therapeutic implications
Source: PLoS One. 2023 May 16;18(5):e0285832. doi: 10.1371/journal.pone.0285832 (PMC10187926; doi:10.1371/journal.pone.0285832)
Supplement: S6 Fig — Evaluation of immunotherapy effect between two subgroups in the GSE91061(A), GSE135222 (B), and VanAllen (C). (DOCX) [file pone.0285832.s006.docx]

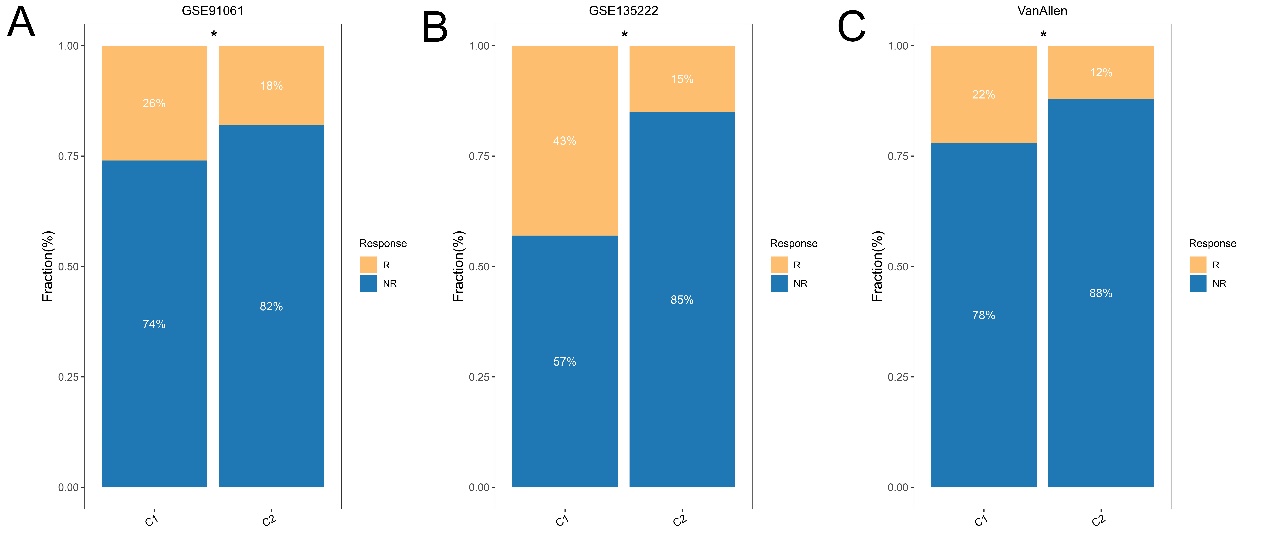


**Figure S6. Evaluation of immunotherapy effect between two subgroups in the GSE91061(A), GSE135222 (B), and VanAllen (C).**
