# Supplementary material for: T cell-mediated tumor killing patterns in head and neck squamous cell carcinoma identify novel molecular subtypes, with prognosis and therapeutic implications
Source: PLoS One. 2023 May 16;18(5):e0285832. doi: 10.1371/journal.pone.0285832 (PMC10187926; doi:10.1371/journal.pone.0285832)
Supplement: S1 File — (ZIP) [file pone.0285832.s008.zip › Supplementary materials/Supplementary Figures.docx]

**Supplementary Figures**

- **Figure S1**
- **Figure S2**
- **Figure S3**
- **Figure S4**
- **Figure S5**
- **Figure S6**


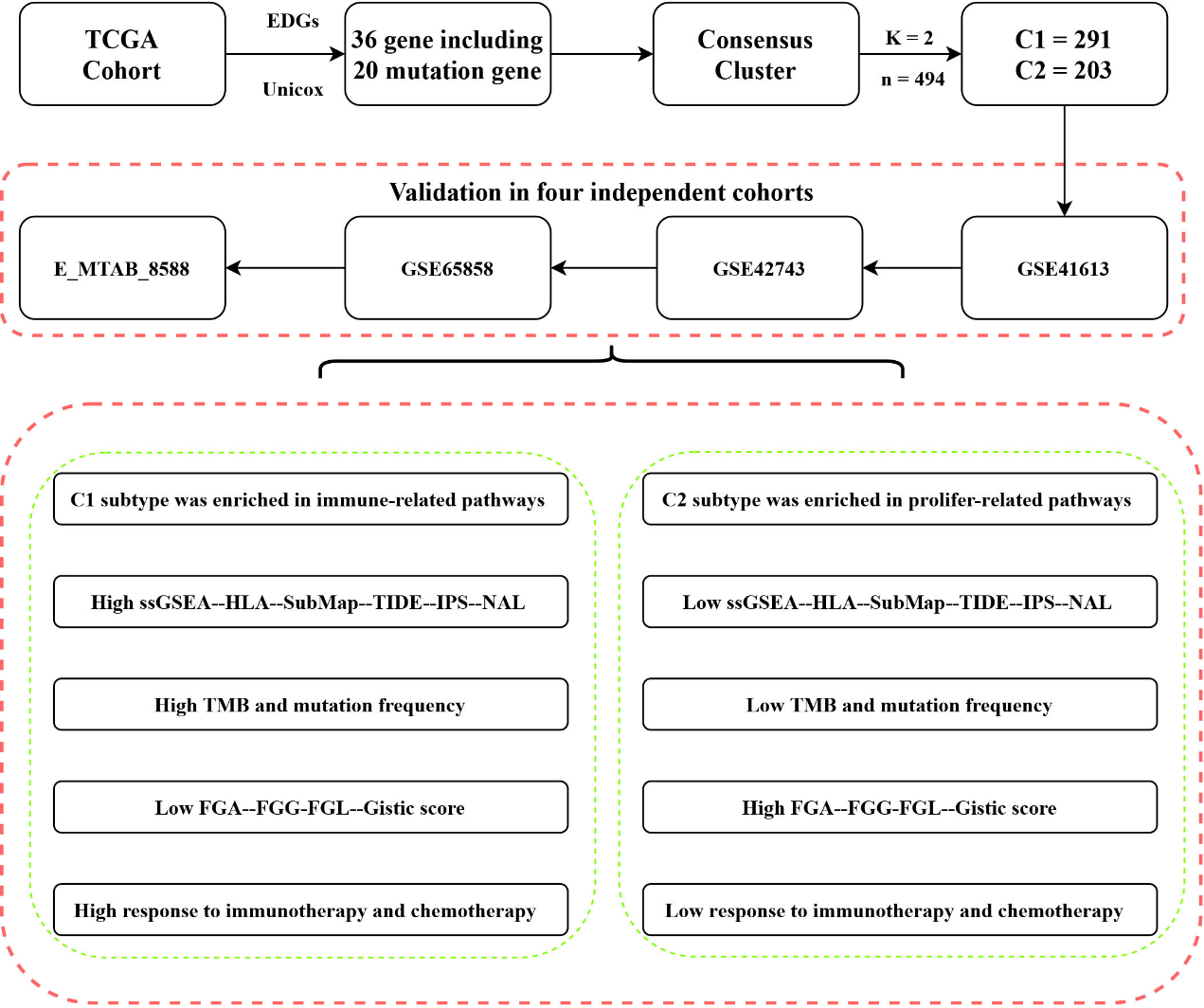


**Figure 1: Flowchart of analysis procedure.**





**Figure S2. CDF curve of Consistent clustering.**


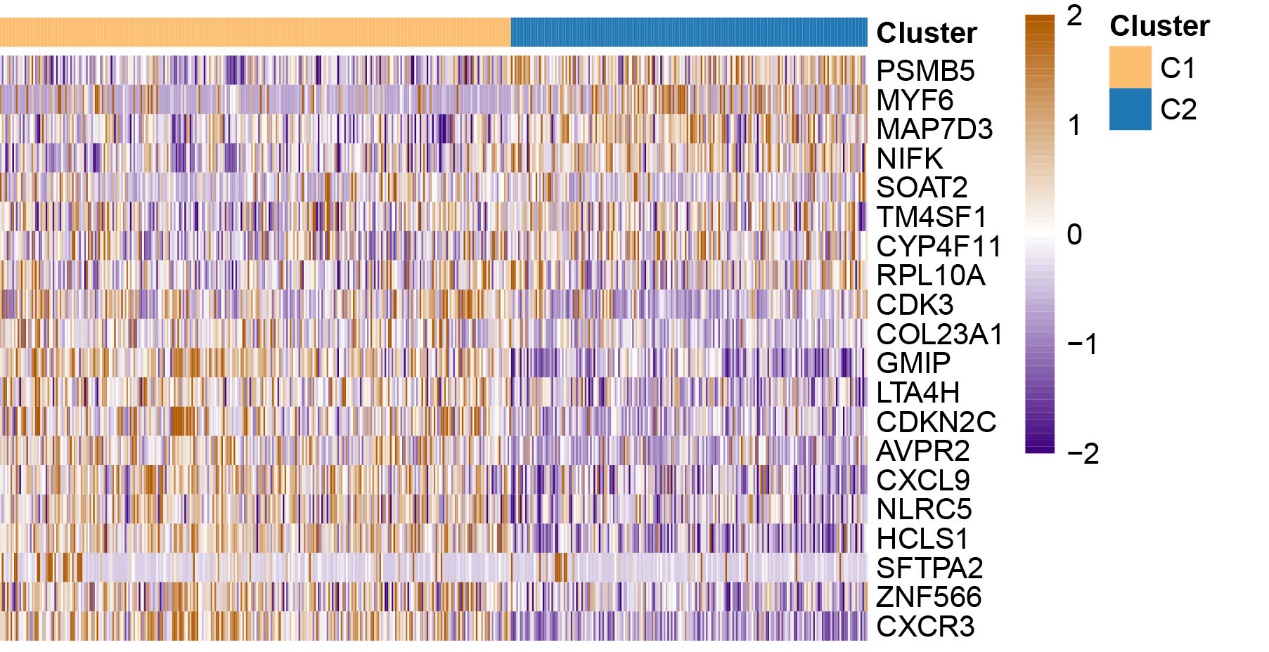


**Figure S3**. **The expression of 20 GSTTKs was displayed by heatmap.**


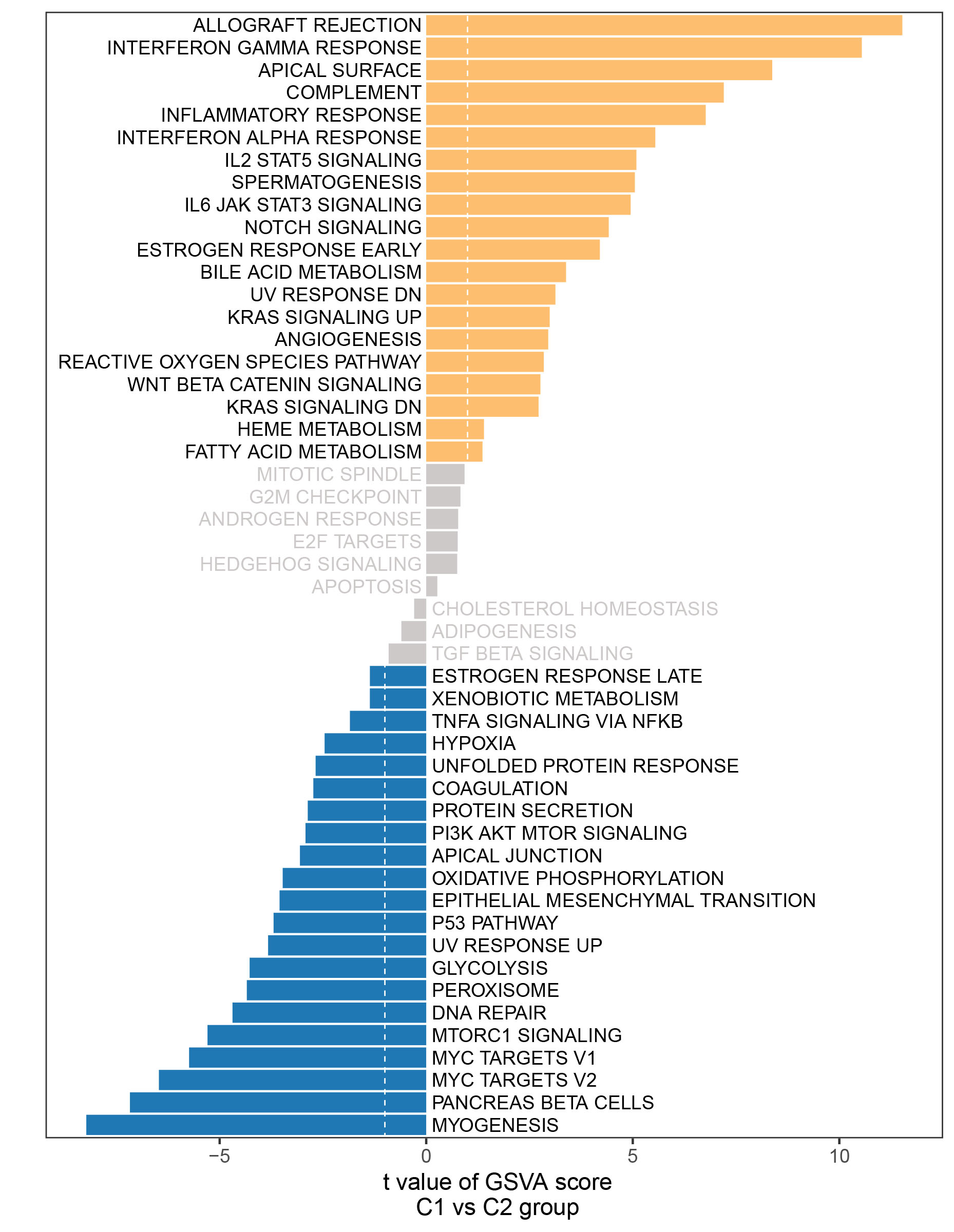


**Figure S4. GSVA enrichment analysis.**


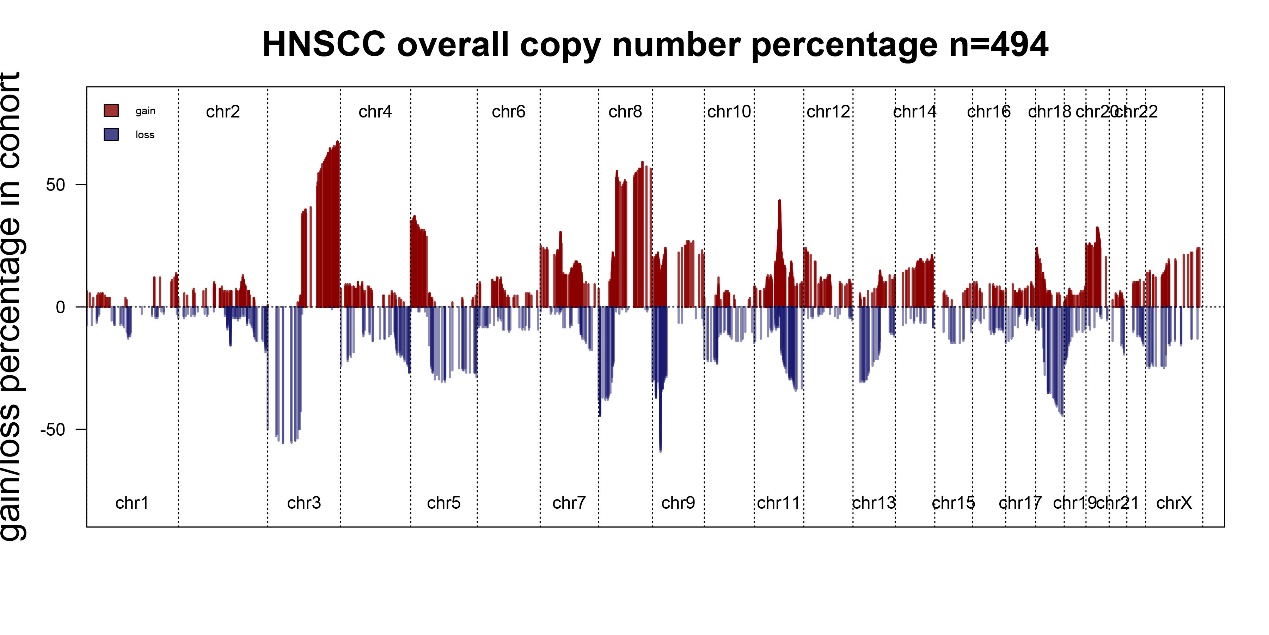


**Figure S5. Copy number percentage of HNSCC patients in TCGA-HNSC cohort.**


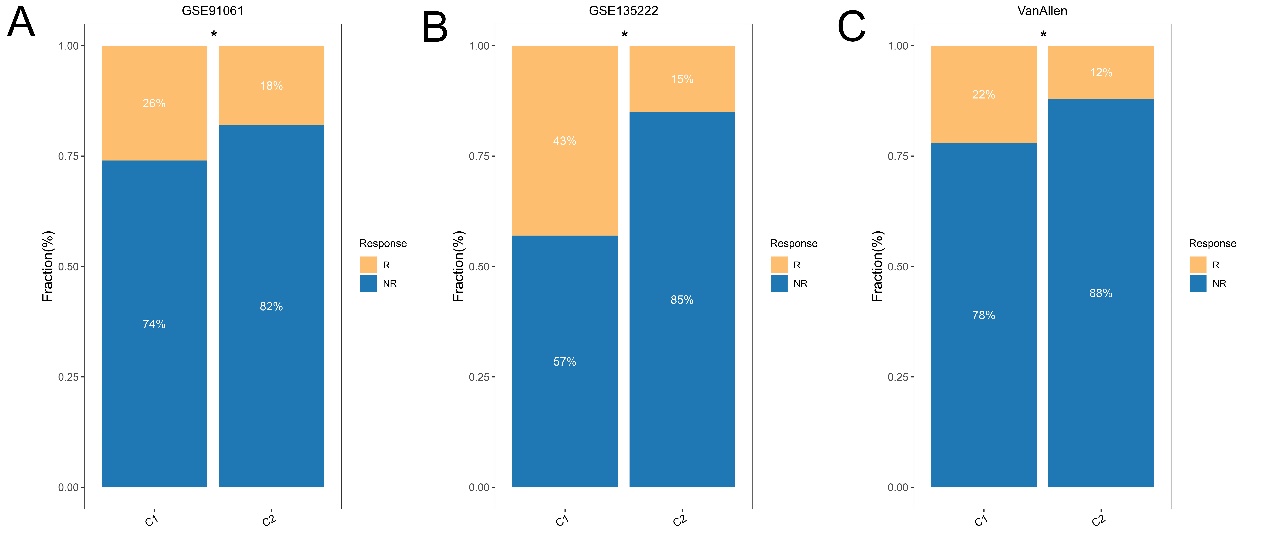


**Figure S6. Evaluation of immunotherapy effect between two subgroups in the GSE91061(A), GSE135222 (B), and VanAllen (C).**
