# Supplementary material for: T cell-mediated tumor killing patterns in head and neck squamous cell carcinoma identify novel molecular subtypes, with prognosis and therapeutic implications
Source: PLoS One. 2023 May 16;18(5):e0285832. doi: 10.1371/journal.pone.0285832 (PMC10187926; doi:10.1371/journal.pone.0285832)
Supplement: S1 File — (ZIP) [file pone.0285832.s008.zip › Supplementary materials/Supplementary materials.docx]

**R package link in Guthub website:**

**edgeR:**

<https://github.com/orgs/channel-mirrors/packages/container/package/bioconda%2Flinux-64%2Fbioconductor-edger>

**VennDiagram:**

<https://github.com/orgs/channel-mirrors/packages/container/package/bioconda%2Flinux-64%2Fr-venndiagram>

**maftools:**

<https://github.com/orgs/channel-mirrors/packages/container/package/bioconda%2Flinux-64%2Fbioconductor-maftools>

**ggplot2:**

<https://github.com/orgs/channel-mirrors/packages/container/package/bioconda%2Flinux-64%2Fr-ggplot2>

**RCircos:**

<https://github.com/orgs/channel-mirrors/packages/container/package/bioconda%2Flinux-64%2Fr-rcircos>

**FactoMineR**

<https://github.com/orgs/channel-mirrors/packages/container/package/conda-forge%2Flinux-64%2Fr-factominer>

**ConsensusClusterPlus:**

<https://github.com/orgs/channel-mirrors/packages/container/package/bioconda%2Flinux-64%2Fbioconductor-consensusclusterplus>

**survival:**

<https://github.com/orgs/channel-mirrors/packages/container/package/conda-forge%2Flinux-aarch64%2Fr-survival>

**WGCNA:**

<https://github.com/orgs/channel-mirrors/packages/container/package/bioconda%2Flinux-64%2Fr-wgcna>

**clusterProfiler:**

<https://github.com/orgs/channel-mirrors/packages/container/package/bioconda%2Flinux-64%2Fbioconductor-clusterprofiler>

**GSVA:**

<https://github.com/orgs/channel-mirrors/packages/container/package/bioconda%2Flinux-64%2Fbioconductor-gsva>

**maftools:**

<https://github.com/orgs/channel-mirrors/packages/container/package/bioconda%2Flinux-64%2Fbioconductor-maftools>
